# Supplementary material for: COZOID: contact zone identifier for visual analysis of protein-protein interactions
Source: BMC Bioinformatics. 2018 Apr 6;19:125. doi: 10.1186/s12859-018-2113-6 (PMC5889581; doi:10.1186/s12859-018-2113-6)
Supplement: Supplementary file 2 — Software build. Executable binary file of the software tool. (ZIP 118,784 kb) [file 12859_2018_2113_MOESM2_ESM.zip › cozoid/java/docs/beansbinding-1.2.1-doc.zip!/org/jdesktop/beansbinding/Binding.SyncFailureType.html]

Binding.SyncFailureType (Beans Binding)


---


|  |  |  |
| --- | --- | --- |
| **Overview** \| **Package** \| **Class** \| **Use** \| **Tree** \| **Deprecated** \| **Index** \| **Help** | |  |
| **PREV CLASS**   **NEXT CLASS** | **FRAMES**    **NO FRAMES**     **All Classes** |  |
| SUMMARY: NESTED \| ENUM CONSTANTS \| FIELD \| METHOD | DETAIL: ENUM CONSTANTS \| FIELD \| METHOD |  |


---


## org.jdesktop.beansbinding Enum Binding.SyncFailureType

```
java.lang.Object
  java.lang.Enum<Binding.SyncFailureType>
      org.jdesktop.beansbinding.Binding.SyncFailureType
```

**All Implemented Interfaces:**: java.io.Serializable, java.lang.Comparable<Binding.SyncFailureType>

**Enclosing class:**: Binding<SS,SV,TS,TV>

---

``` public static enum Binding.SyncFailureType extends java.lang.Enum<Binding.SyncFailureType> ```

An enumeration representing the reasons a sync (`save` or `refresh`)
can fail on a `Binding`.

**See Also:**: `Binding.refresh()`, `Binding.save()`

---

**Enum Constant Summary**

`CONVERSION_FAILED`
  
          A `save` failed due to a conversion failure on the value
returned by the `Binding's` target property for the `Binding's`
target object.

`SOURCE_UNREADABLE`
  
          A `refresh` failed because the `Binding's` source property is unreadable
for the `Binding's` source object.

`SOURCE_UNWRITEABLE`
  
          A `save` failed because the `Binding's` source property is unwriteable
for the `Binding's` source object.

`TARGET_UNREADABLE`
  
          A `save` failed because the `Binding's` target property is unreadable
for the `Binding's` target object.

`TARGET_UNWRITEABLE`
  
          A `refresh` failed because the `Binding's` target property is unwriteable
for the `Binding's` target object.

`VALIDATION_FAILED`
  
          A `save` failed due to a validation failure on the value
returned by the `Binding's` target property for the `Binding's`
target object.


| **Method Summary** | |
| --- | --- |
| `static Binding.SyncFailureType` | `valueOf(java.lang.String name)`             Returns the enum constant of this type with the specified name. |
| `static Binding.SyncFailureType[]` | `values()`             Returns an array containing the constants of this enum type, in the order they are declared. |

**Methods inherited from class java.lang.Enum**

`clone, compareTo, equals, finalize, getDeclaringClass, hashCode, name, ordinal, toString, valueOf`

**Methods inherited from class java.lang.Object**

`getClass, notify, notifyAll, wait, wait, wait`

**Enum Constant Detail**

### TARGET\_UNWRITEABLE

```
public static final Binding.SyncFailureType TARGET_UNWRITEABLE
```

:   A `refresh` failed because the `Binding's` target property is unwriteable
    for the `Binding's` target object.

---


### SOURCE\_UNWRITEABLE

```
public static final Binding.SyncFailureType SOURCE_UNWRITEABLE
```

:   A `save` failed because the `Binding's` source property is unwriteable
    for the `Binding's` source object.

---


### TARGET\_UNREADABLE

```
public static final Binding.SyncFailureType TARGET_UNREADABLE
```

:   A `save` failed because the `Binding's` target property is unreadable
    for the `Binding's` target object.

---


### SOURCE\_UNREADABLE

```
public static final Binding.SyncFailureType SOURCE_UNREADABLE
```

:   A `refresh` failed because the `Binding's` source property is unreadable
    for the `Binding's` source object.

---


### CONVERSION\_FAILED

```
public static final Binding.SyncFailureType CONVERSION_FAILED
```

:   A `save` failed due to a conversion failure on the value
    returned by the `Binding's` target property for the `Binding's`
    target object.

---


### VALIDATION\_FAILED

```
public static final Binding.SyncFailureType VALIDATION_FAILED
```

:   A `save` failed due to a validation failure on the value
    returned by the `Binding's` target property for the `Binding's`
    target object.


**Method Detail**

### values

```
public static Binding.SyncFailureType[] values()
```

:   Returns an array containing the constants of this enum type, in
    the order they are declared. This method may be used to iterate
    over the constants as follows:

    ```
    for (Binding.SyncFailureType c : Binding.SyncFailureType.values())
        System.out.println(c);
    ```

    :   **Returns:**: an array containing the constants of this enum type, in the order they are declared

---


### valueOf

```
public static Binding.SyncFailureType valueOf(java.lang.String name)
```

:   Returns the enum constant of this type with the specified name.
    The string must match *exactly* an identifier used to declare an
    enum constant in this type. (Extraneous whitespace characters are
    not permitted.)

    :   **Parameters:**: `name` - the name of the enum constant to be returned. **Returns:**: the enum constant with the specified name **Throws:**: `java.lang.IllegalArgumentException` - if this enum type has no constant with the specified name: `java.lang.NullPointerException` - if the argument is null


---


|  |  |  |
| --- | --- | --- |
| **Overview** \| **Package** \| **Class** \| **Use** \| **Tree** \| **Deprecated** \| **Index** \| **Help** | |  |
| **PREV CLASS**   **NEXT CLASS** | **FRAMES**    **NO FRAMES**     **All Classes** |  |
| SUMMARY: NESTED \| ENUM CONSTANTS \| FIELD \| METHOD | DETAIL: ENUM CONSTANTS \| FIELD \| METHOD |  |


---
